# EasyClick: an improved system for confocal microscopy of live roots with a user-optimized sample holder

**DOI:** 10.1007/s00425-023-04293-y

**Published:** 2023-12-14

**Authors:** Kateřina Kaduchová, Vratislav Čmiel, Veronika Koláčková, Ales Pecinka

**Affiliations:** 1 Centre of Plant Structural and Functional Genomics, Institute of Experimental Botany of the Czech Academy of Sciences, Šlechtitelů 31, 779 00 Olomouc, Czech Republic; 2 Department of Cell Biology and Genetics, Faculty of Science, Palacký University, 779 00 Olomouc, Czech Republic; 3 Department of Biomedical Engineering, Faculty of Electrical Engineering and Communication, Brno University of Technology, Technická 3082/12, 61600 Brno, Czech Republic

**Keywords:** Barley, Confocal microscopy, EasyClick, Growth, *Hordeum vulgare*, Live cell imaging, Plant, Root

## Abstract

**Main conclusion:**

We describe a user-optimized sample holder EasyClick for medium-sized plants that reduces root side movements and thus greatly extends the duration of live cell confocal microscopy.

**Abstract:**

Preparation and mounting of the samples are key factors for successful live cell microscopy. To acquire biologically relevant data, it is necessary to minimize stress and avoid physical damage to plant tissues during the installation of the sample into the microscope. This is challenging, particularly when the whole plant is mounted as the living sample needs to be properly anchored in the microscopic system to obtain high-quality and high-resolution data. Here, we present a user-optimized sample holder EasyClick for live cell inverted confocal microscopic analysis of plant roots with diameters from 0.3 to 0.7 mm. The EasyClick holder was tested on an inverted confocal microscope using germinating plants of several cereals. Nevertheless, it can be directly used on other types of inverted microscopes from various producers and on different plant species. The EasyClick holder effectively restricts root lateral and vertical movements. This greatly improves the conditions for time-lapse microscopy of the samples of interest.

## Introduction

High-quality live cell microscopy is essential for understanding the principles of plant cell biology (Nagy et al. 2012; Hamant et al. 2019). At the confocal (180 nm lateral and 500 nm axial) resolution, mutual cell–cell, cell–molecule and/or molecule–molecule interactions can be explored (Fouquet et al. 2015; Elliott 2020). Compared to confocal in vivo analysis of animal cell cultures or early developmental stages of small-to-middle sized animal models (e.g., *Drosophila melanogaster, Danio rerio*), plant researchers are facing a variety of difficulties when setting experiments, including strong autofluorescence of chlorophyll and secondary metabolites, interference of the cell walls and plant growth affected by gravitropism. The gravity-directed growth controls both the upward growth of the shoot and leaves and the downward growth of roots, enabling the proper uptake of water and ions required for plant development (Chen et al. 1999). Due to gravitropism, plants placed in a non-vertical direction can quickly react and point their growth in the vertical direction again. As amyloplasts are not fixed in the root cap cells, they sprinkle frequently during growth which makes the root rotate and twist along the elongation axis (Rahni and Birnbaum 2019). In standard confocal microscopes, the sample is placed horizontally during the scanning. When the living plant is set between glass slides, the root immediately reacts to rotate. These moves regularly reach a growth rate 120–800 μm h^−1^ in various model plants, e.g., 120–150 μm h^−1^ in *Arabidopsis thaliana*, ~ 700 μm h^−1^ in barley (*Hordeum vulgare*) or ~ 800 μm h^−1^ in rice (*Oryza sativa*) (Yazdanbakhsh and Fisahn 2010; Higuchi et al. 2017). Taken together, the stochastic root movements make live cell microscopy of the root apical meristems challenging.

There are several approaches how to prevent or restrict excessive root movements on a microscopic slide. Root movements in the x–y-axis leading to root twisting were previously decreased by placing the plant between two nylon strings fixed on a slide or into the microscopic slide chambers used for cell culture chemical treatments (Grossmann et al. 2011; Rahni and Birnbaum 2019). The movement in the z-axis is normally restricted by covering the sample with a coverslip but sometimes this limitation is not enough, especially when the roots with a wider diameter are analyzed. In some setups, the coverslip can be replaced by the agarose block placed on the top of the lying root which pushes it equally down onto a slide surface (Rahni and Birnbaum 2019).

Even though all these approaches improved plant root microscopy quality, the preparation of the plant sample itself is time-consuming and not user-friendly in these systems. To increase sample preparation effectiveness and enable long-term microscopy scanning, we co-developed a custom-optimized microscopy holder EasyClick facilitating plant root analysis. The tested version of the holder is primarily designed for the microscopy of plants with a root diameter from 0.3 to 0.7 mm, which includes, e.g., several cereal crops such as barley, wheat, or rye.

## Materials and methods

### Rapid movements of barley roots in a standard microscopy setup

**Fig. 1:**
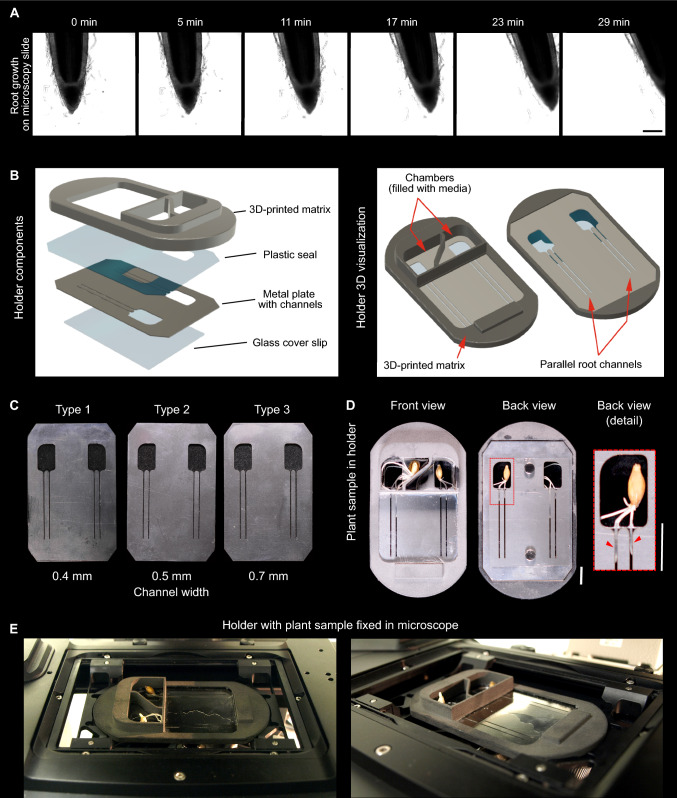
Barley root movements during time-lapse scanning and description of the microscopy EasyClick holder. **A** Time-lapse of barley free root growth on a microscopy slide using transmission light microscopy. Total scanning time 29 min. Scale bar = 200 μm. **B** 3D model of individual EasyClick parts. **C** Three types of the metal plate differing in the growth channel widths (0.4, 0.5 and 0.7 mm). **D** Detailed view of germinating barley plants in the holder. Scale bars = 1 cm. **E** Installation of the EasyClick holder in the inverted microscope

Recently, we established a series of barley fluorescent marker lines for monitoring nuclear compartments and microtubules (Kaduchová et al. 2023). During this analysis, we noted rapid movements of barley root apices in the frontal, lateral, and vertical directions. This had a major negative impact on the duration of individual scanning windows and the identification of the cells (Fig. 1A). Due to these movements, a cell or mitotic division of interest frequently appeared out of the focus or even the scanned area. To mitigate this issue, we tested a prototype of a microscopy holder named EasyClick (Pragolab s.r.o., Praha, Czech Republic; catalog numbers of individual parts: VIV0004, VIV0013, T220007, T220008, T220009) that was designed to reduce root movements by defining the growth direction and decreasing the probability of root damage during the mounting.

### Preparation of the plant sample

The initial optimization was done using the wild-type two-row spring barley (*Hordeum vulgare* L.) cultivar Golden Promise (PI 343079; from the National small grains collection of the national plant germplasm system of the United states department of agriculture-agricultural research service) and fluorescent marker line ENHANCED YELLOW FLUORESCENT PROTEIN—HISTONE 2B (EYFP-H2B) in Golden Promise background (Kaduchová et al. 2023). Dry barley seeds were stratified on the wet filter paper soaked in tap water at 4 °C for 48 h in the dark and then transferred to 24 °C for 48 h to germinate in the dark.

### Mounting of the sample into the EasyClick microscopy holder

The principle of the EasyClick microscopy holder components and their assembly are shown in Fig. 1B–D. The EasyClick consists of a three-dimensional (3D)-printed plastic holder matrix (PA11 material, powder laser sintering SLS) with embedded magnets on both sides, a stainless steel metal plate (food and gastronomy industry use, 0.5 mm thickness) with 37 mm long growth channels of different widths, a 0.5 mm thick polymethyl methacrylate plastic seal, and a large coverslip (60 × 45 mm; Karl Hecht, Sondheim vor der Rhön, Německo) (Fig. 1B–D). 3D-printed holder matrix was permanently fixed with the plastic seal (quick drying glue; Kavan, Pardubice, Czech Republic). The remaining parts (metal plate, coverslip) are removable and fixed via magnets (Fig. 1D).

During the fixation of the sample, the metal plate was put on the backside of the 3D-printed matrix on the plastic seal where it was held with magnets. In the second step, a few drops of liquid growth media were dripped on the metal surface. After covering the metal plate with a large coverslip, the liquid media filled the metal plate channels. The large cover slip was afterwards fixed on the metal plate with magnets. Subsequently, the plant sample was placed into one of two positions bordered by raised holder edges making small 18 × 41 × 6 mm plant containers (Fig. 1D). The construction of the EasyClick metal plate with ground edges allowed for easy penetration of the root into the selected channel. After setting plants in proper positions, the containers were filled with liquid growth media, alternatively, containers can be covered with a wet tissue to reduce transpiration. A liquid was refilled with a Pasteur pipette during the time-lapse experiments without any plant growth disruption and microscopy stage movements. The EasyClick holder is easily fitted into the universal microscope stage mounting system (Fig. 1E). Microscopic images were acquired using a Leica TCS SP8 STED3X confocal microscope (Leica Microsystems, Wetzlar, Germany), equipped with an HC PL APO CS2 20x/0.75 DRY and HC PL APO CS2 639/1.40 Oil objective, hybrid detectors (HyD), and the Leica Application Suite X (LAS-X) software version 3.5.5 with the Leica Lightning module (Leica, Buffalo Grove, IL, USA). Time-lapse confocal Z-stack images of EYFP-H2B of approximately 20–30 µm width were captured using 508 nm laser excitation line and appropriate emission spectrum in 4-min intervals. Single time-lapse scans of roots in transmission light were captured in 1-min intervals. Images were processed in Adobe pre-hybridize shop version 12.0 (Adobe Systems), Imaris version 9.7.2 (Oxford Instruments), and Inkscape (Inkscape project).

Three different custom-optimized variants of the metal plates (Type 1–3), differing in the growth channel widths (0.4 mm; 0.5 mm, and 0.7 mm), were used depending on the size of the roots of the analyzed plant (Fig. 1D). For each metal plate, up to two roots of two different plant samples were installed and analyzed in short time intervals (four roots per metal plate in total). More than 4 cm length of the channels enables the time-lapse scanning theoretically of more than 48 h (counted for root growth rate of 600 μm h^−1^ when 1 cm long root is placed into 4 cm channel). However, we recommend to use young (1 or 2 days old) seedlings with roots approximately 1–2 cm long for the microscopy analysis. Such roots can be easily placed into the holder channel without a risk of damage caused by their length and can use the whole channel length for growth. Due to the non-transparent material of the metal plate, we recommend first navigating the root tip in the channel directly to the light path of the objective and only then focusing at the Z-axis.

## Results

**Fig. 2:**
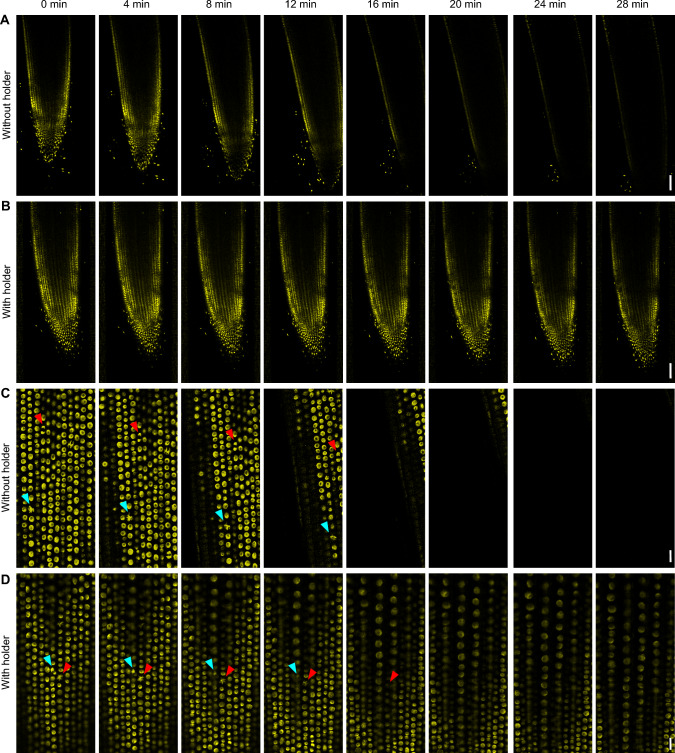
Live cell confocal microscopy (CLSM) imaging of root apical meristems without and with EasyClick holder. **A** Representative free growth of a root from 2 days old barley plant without holder. Nuclei and chromosomes were visualized with the chromatin marker eYFP-H2B. Scanning time = 28 min. Scale bar = 100 μm. **B** Channel-directed root movement with EasyClick holder using the same fluorescent marker line as in (**A**). Scale bar = 100 μm. **C** Detailed time-lapse single-cell layer scanning of root growing on a microscopy slide without the holder. Note the shifts in both the horizontal (red arrowhead) and vertical (cyan arrowhead) directions. Arrowheads of the same color mark particular cell tracked in time. Scale bar = 10 μm. **D** Situation as in **C** with improved parameters due to EasyClick holder use. Arrowheads of the same color mark particular cell tracked in time. Scale bar = 10 μm

During the microscopy analysis, the root movements in the y-axis (axis perpendicular to the root growth axis) are limited by channel walls. Channel width should be selected so that it leads the root straight while providing enough space for its growth (Fig. 1B–D). A test 28 min scanning revealed that the root grew out of the analysis field under standard settings (microscopy slide without any root restrictions) nevertheless the root placed in the EasyClick microscopy holder remained in the scanning field and could still be analyzed even after this scanning time (Fig. 2, red arrowheads).

Similarly, the EasyClick channels reduce root twisting in the z-axis due to the thickness of the metal plate (500 μm), roughly corresponds to the diameter of wider barley roots. This enables root growth in the proximity of the coverslip in a limited space. Despite the optimized channel z-axis height, it is still challenging to precisely set a root cell layer for detailed microscopy analysis where the focal plane and vertical position of the object of interest will not change in time. Furthermore, the conically shaped meristematic zone represents the most problematic part of the root in our microscopy analysis. As the root grows, the cells in this region are pushed into the higher scanning layers due to the production of new cells via mitotic divisions (Fig. 2C,D; cyan arrowheads). This may even lead to a change of the focus plane from epidermal cell layer (Fig. 2D, 0–12 min) to the first cortex cell layer (Fig. 2D, 16–28 min). The holder cannot solve completely this problem but reduces it by stabilizing the samples in several directions.

**Fig. 3:**
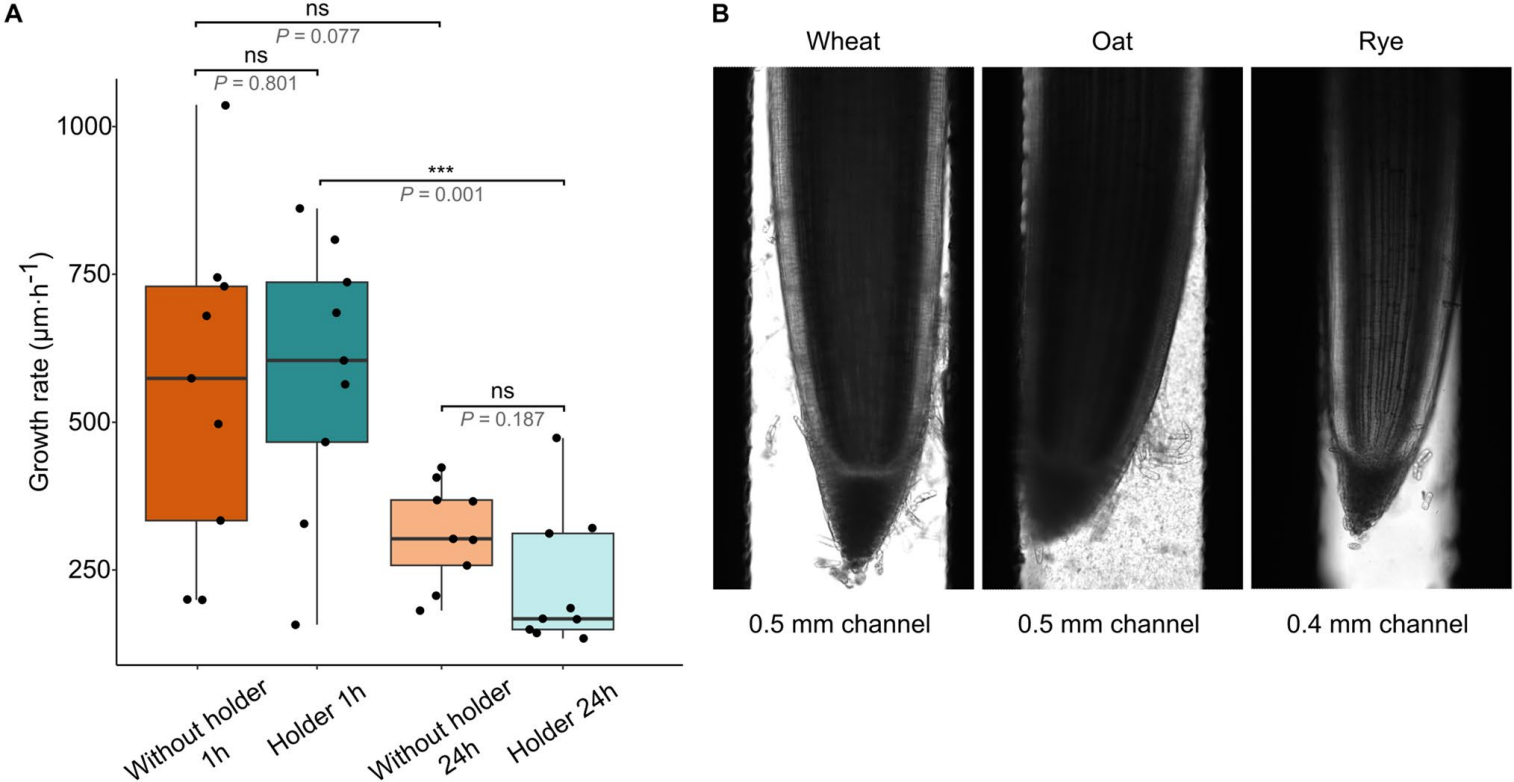
Root growth rate and mounting of roots from various cereals. **A** Roots growth rate from 2 days old barley plants grown freely on a microscopic slide (without holder 1 h; *n* = 9 roots), shortly after introduction into the EasyClick (holder 1 h; *n* = 9 roots), after 24 h of freely growing on a microscopy slide (without holder 24 h; *n* = 9), and after 24 h in the EasyClick (holder 24 h; *n* = 9). The total recording time of 32 min and the values were transformed to 1 h of total growth time. The horizontal black lines within the boxes mark the median, the lower and upper hinges of the boxplots correspond to the first and third quartiles of the data, and the whiskers mark 10 and 90% intervals. Asterisks above the boxes indicate statistically significant differences. The significance level = 0.05, Kruskal–Wallis test with post-hoc Dunn’s test, *P* = 0.002. Scale bar = 0.2 mm. **B** Examples of roots of other cereals introduced into the EasyClick system. For the roots of wheat and oat plants, we used Type 2 metal plate (0.5 mm channels) and for the roots of rye, we used Type 1 plate (0.4 mm channels)

Finally, we assessed whether the EasyClick holder affects barley root growth along its main growth axis (Fig. 3A). We compared the root growth rate of unrestricted roots placed on a microscopy slide (without holder) and roots placed in a microscopy holder during the 32-min time-lapse scanning. For better clarity, we calculated the root growth rate for a reference time of 1 h. There was a greater variability as to the root growth rate in unrestricted conditions on the microscopy slide, but the median growth rate was almost the same (*P* = 0.801) for both conditions (in holder 604.1 μm h^−1^; without holder on slide 573.7 μm h^−1^). Afterward, we measured the growth rate of roots after 24 h in the holder and on a microscopy slide. Although the median root growth rate of samples from the holder (167.3 μm h^−1^) and on a microscopy slide (302.8 μm h^−1^) differed by 1.8-fold, it was not significant (*P* = 0.187), possibly due to a relatively large variation between individual roots (Fig. 3A). When comparing the growth rate between 1 and 24 h setups, the reduction was not significant for the free growing roots (*P* = 0.077), but opposite was true for the roots in holder (*P* = 0.001; Fig. 3A).

Finally, we tested the versatility of the EasyClick holder for root microscopy of different crops (Fig. 3B). The Type 1 metal plate (0.4 mm channel width) fitted for the rye (*Secale cereale*), and Type 2 metal plate (0.5 mm channel width) for the barley, wheat (*Triticum aestivum*), and oat (*Avena sativa*). The Type 3 metal plate (0.7 mm channel width) was ideal for some of the oat and wheat roots with a wider diameter. We also attempted to test the germinating roots of several other crops such as faba bean (*Vicia faba*), garden pea (*Pisum sativum*) and maize (*Zea mays*), but their roots were too thick even for the Type 3 plate (not shown).

## Discussion

Several systems for monitoring the growth of living plant roots in microscopes were already described and can improve imaging of various plants as demonstrated for e.g., *Arabidopsis thaliana* and *Medicago truncatula* (Grossmann et al. 2011; Ovecka et al. 2015; Berthet and Maizel 2016; von Wangenheim et al. 2017). Specialized systems have been engineered to explore interactions between plant roots and microbes or worms (Parashar and Pandey 2011; Vernet et al. 2022).

The systems can be divided into two main groups based on the direction of plant root cultivation: (i) vertical and (ii) horizontal. The vertical systems frequently utilize either light-sheet microscopy technology and the samples are mounted in different types of capillaries (Ovecka et al. 2015; Vernet et al. 2022) or less often also confocal microscopy (von Wangenheim et al. 2017). The vertical confocal systems are typically based on microscopes that are turned 90° to bring the slides with samples in a vertical position (von Wangenheim et al. 2017). The advantage of these systems is that the roots can growth more-or-less straight towards the center of gravity as typical for many plants (Chen et al. 1999; Migliaccio et al. 2013). The other main direction of systems forces plants to grow in a horizontal position. These systems are explored for various complex setups combining microscopy with microfluidic systems (Grossmann et al. 2011; Parashar and Pandey 2011) or simply when the access to vertical systems is not available (Rahni and Birnbaum 2019). The EasyClick system represents the latter setup and is so far the only system that was tested and successfully used to on temperate cereals (Kaduchová et al. 2023).

The horizontal growth of the plants is an obvious limitation for studies where root gravitropism is essential for understanding given processes. It also has to be noted that not all roots under natural conditions grow straight down and that soil contains various objects (e.g., stones) that can cause roots to grow horizontally for some time until the object can be bypassed. Therefore, the tendency of the root to bend towards the center of gravity might represent a rather technical than biological problem. For applications where vertical growth is needed, the EasyClick system could possibly be adapted for mounting into a 90° turned confocal microscope (von Wangenheim et al. 2017). Alternatively, an add-on on horizontal microscopes allowing observations in a vertical manner such as GraviKit could be applied (Feldhaus et al. 2021).

The forward and to some extent also side movements of the conically shaped root tips represents a major challenge for live cell microscopy analysis because the cells of interest dynamically change their position in all directions. Therefore, it is essential to precisely fix the living sample in space for a certain time while simulating more-or-less natural conditions for its growth. Our initial trials to lead barley roots between two stretched fishing strings fixed on a microscopy glass as described (Rahni and Birnbaum 2019), or covering the root with a block of agar did not work well due to its thickness. Therefore, we tested the EasyClick microscopy holder, which guides roots through narrow channels without disturbing their growth. There were no significant differences in the rate of root growth freely on the microscopic slide versus EasyClick at 1 h and then after 24 h. However, this parameter is relatively highly variable between individual roots which could mask some subtle trends. While there were no significant differences between mounting methods, there was a clear reduction in the root growth rate after 24 h for both approaches and the difference was even statistically significant for the holder. The cause of this is unknown and we could not find data for barley root growth rate in specific types of soil. We hypothesize that barley roots might grow slower in soil than in free space on microscopic slides and that they also naturally reduce their growth rate over time.

The great advantage of this system is a quick and easy assembly minimizing plant damage and reducing stress. As the EasyClick holder permits plants to be grown for several hours or even days, it is possible to firstly set two plants into the holder, let them grow for a distinct time to overcome potential manipulation stress, and then apply some highly-sensitive analyses like microtubule or chromosome dynamics (Kaduchová et al. 2023). Using of different growth media will also allow for assessing mid- and long-term effects of specific nutrients, treatments, or stresses. However, the growth compartments on the tested EasyClick model were not separated by a watertight wall which means that only one type of growth media can be applied at the time. The system can be used practically for germinating plants of a wide range of species with root diameters below 0.7 mm. We demonstrated it by using EasyClick in combination with different cereal species. However, the current set of metal plates might be exclusive for species with larger roots such as e.g., garden pea, faba bean or maize. Their roots were too thick even for the Type 3 metal plate.

One limitation noted in our analyses was the conical shape of the root tip. When focused on a rhizodermal layer close to the tip, the frontal proliferation of the root and its thickening caused a switch of the focus to cortex cell layer(s) and the disappearance of the initially monitored cells. There are several possible solutions. First, using a lower magnification with a greater focal depth. Second, extending the size of the z-stack. However, this might lead to longer scanning times and exposure of plant cells to the laser. Finally, the microscopy could focus on a position more distal from the root tip that already underwent the expansion and will not change much anymore.

In conclusion, the tested 3D-printed EasyClick microscopy holder is a useful addition to the microscopy toolbox for live cell confocal imaging within the roots of plants with root diameter from 0.3 to 0.7 mm. The holder can help plant researchers perform precise confocal microscopy analysis in a user-friendly manner. Moreover, this microscopy holder enables a high-quality analysis on a wide range of confocal microscopes, where the sample is placed horizontally during the scanning in the majority of cases. We envision future development of the upright version of the EasyClick holder that will extend its use also to non-inverted confocal microscopes.

## Data Availability

Data are available within the article.

## Abbreviations

3D: Three dimensional
EYFP: Enhanced yellow fluorescent protein
H2B: HISTONE 2B

## References

[CR1] Berthet B, Maizel A (2016). Light sheet microscopy and live imaging of plants. J Microsc.

[CR2] Chen RJ, Rosen E, Masson PH (1999). Gravitropism in higher plants. Plant Physiol.

[CR3] Elliott AD (2020). Confocal microscopy: principles and modern practices. Curr Protoc Cytom.

[CR4] Feldhaus C, Kolb M, Küppers M, Hardy S, Palmisano R (2021). GraviKit: An easy-to-implement microscope add-on for observation of gravitation dependent processes. (preprint). Biorxiv.

[CR5] Fouquet C, Gilles JF, Heck N, Dos Santos M, Schwartzmann R, Cannaya V, Morel MP, Davidson RS, Trembleau A, Bolte S (2015). Improving axial resolution in confocal microscopy with new high refractive index mounting media. PLoS ONE.

[CR6] Grossmann G, Guo WJ, Ehrhardt DW, Frommer WB, Sit RV, Quake SR, Meier M (2011). The RootChip: An integrated microfluidic chip for plant science. Plant Cell.

[CR7] Hamant O, Das P, Burian A, Cvrčková F, Žárský V (2019). Time-lapse imaging of developing shoot meristems using a confocal laser scanning microscope. Plant cell morphogenesis. Methods in molecular biology.

[CR8] Higuchi K, Ono K, Araki S, Nakamura S, Uesugi T, Makishima T, Ikari A, Hanaoka T, Sue M (2017). Elongation of barley roots in high-pH nutrient solution is supported by both cell proliferation and differentiation in the root apex. Plant Cell Environ.

[CR9] Kaduchová K, Marchetti C, Ovečka M, Galuszka P, Bergougnoux V, Šamaj J, Pecinka A (2023). Spatial organization and dynamics of chromosomes and microtubules during barley mitosis. Plant J.

[CR10] Migliaccio F, Tassone P, Fortunati A (2013). Circimnutation as an autonomous root movement in plants. Am J Bot.

[CR11] Nagy G, Kiraly G, Banfalvi G (2012) Optimization of cell cycle measurement by time-lapse microscopy. In: Conn PM (ed) Laboratory methods in cell biology. Methods in cell biology 112:143–161. doi:10.1016/b978-0-12-405914-6.00007-x

[CR12] Ovecka M, Vaskebova L, Komis G, Luptovciak I, Smertenko A, Samaj J (2015). Preparation of plants for developmental and cellular imaging by light-sheet microscopy. Nat Protoc.

[CR13] Parashar A, Pandey S (2011). Plant-in-chip: microfluidic system for studying root growth and pathogenic interactions in *Arabidopsis*. Appl Phys Lett.

[CR14] Rahni R, Birnbaum KD (2019). Week-long imaging of cell divisions in the Arabidopsis root meristem. Plant Methods.

[CR15] Vernet H, Fullana AM, Sorribas FJ, Gualda EJ (2022). Development of microscopic techniques for the visualization of plant-root-knot nematode interaction. Plants-Basel.

[CR16] von Wangenheim D, Hauschild R, Fendrych M, Barone V, Benkova E, Friml J (2017). Live tracking of moving samples in confocal microscopy for vertically grown roots. Elife.

[CR17] Yazdanbakhsh N, Fisahn J (2010). Analysis of *Arabidopsis thaliana* root growth kinetics with high temporal and spatial resolution. Ann Bot.

